# The nucleolar protein GLTSCR2 is required for efficient viral replication

**DOI:** 10.1038/srep36226

**Published:** 2016-11-08

**Authors:** Peng Wang, Wen Meng, Shi-Chong Han, Cui-Cui Li, Xiao-Jun Wang, Xiao-Jia Wang

**Affiliations:** 1 Key Laboratory of Zoonosis of Ministry of Agriculture, China Agricultural University, Beijing, China; 2 State Key Laboratory of Veterinary Biotechnology, the Chinese Academy of Agriculture Sciences, Harbin, China

## Abstract

Glioma tumor suppressor candidate region gene 2 protein (GLTSCR2) is a nucleolar protein. In the investigation of the role of GLTSCR2 that played in the cellular innate immune response to viral infection, we found GLTSCR2 supported viral replication of rhabdovirus, paramyxovirus, and coronavirus in cells. Viral infection induced translocation of GLTSCR2 from nucleus to cytoplasm that enabled GLTSCR2 to attenuate type I interferon IFN-β and support viral replication. Cytoplasmic GLTSCR2 was able to interact with retinoic acid-inducible gene I (RIG-I) and the ubiquitin-specific protease 15 (USP15), and the triple interaction induced USP15 activity to remove K63-linked ubiquitination of RIG-I, leading to attenuation of RIG-I and IFN-β. Blocking cytoplasmic translocation of GLTSCR2, by deletion of its nuclear export sequence (NES), abrogated its ability to attenuate IFN-β and support viral replication. GLTSCR2-mediated attenuation of RIG-I and IFN-β led to alleviation of host cell innate immune response to viral infection. Our findings suggested that GLTSCR2 contributed to efficient viral replication, and GLTSCR2 should be considered as a potential target for therapeutic control of viral infection.

Innate immunity is critical for defending the host from pathogens, and type I interferon (IFN) is the core of cellular antiviral response^1^,^2^,^3^,^4^,^5^. Upon viral infection, host pattern recognition receptors (PRRs), such as the retinoic acid-induced gene I (RIG-I)-like receptors (RLRs), are able to detect viral nucleic acids and initiate a series of cell signals, leading to induction of type I IFN and proinflammatory cytokines^2^,^3^,^4^,^5^. RIG-I, a family member of RLRs, interacts with viral RNA and recruits mitochondrial-associated virus stimulator (MAVS, also known as IPS-1, Cardif, and VISA)^6^,^7^. MAVS recruits tumor necrosis receptor-associated factor 3 (TRAF3) that results in TRAF3 lysine 63 (K63)-linked auto-ubiquitination to provide docking sites for TANK binding kinase 1/I kappa-B kinase epsilon (TBK1/IKKε) complex^8^,^9^,^10^. This complex undergoes auto-phosphorylation-mediated activation, resulting in phosphorylating and activating the type I IFN regulatory factor IRF3 or/and IRF7 to form homodimers or heterodimers that translocate into the nucleus for induction of type I IFN^10^,^11^. The complex also activates canonical IKK for NF-κB activation to induce proinflammatory cytokines^12^. Studies also showed that dephosphorylation (Thr170) of RIG-I by the phosphatase PP1 α/γ^13^ and K63-linked ubiquitination (Lys172) of RIG-I by the ubiquitin E3 ligase tripartite motif proteins TRIM25^14^ and TRIM4^15^ lead to RIG-I activation and type I IFN production. Studies have shown that viral infection, including rhabdovirus, paramyxovirus, coronavirus, and herpesvirus, may counteract RIG-I-dependent IFN antiviral response^16^. Rabies virus is a member of the *Rhabdoviridae* family, and Rabies virus (Ni strain)-expressed N protein has a function to evade the activation of RIG-I and RIG-I-mediated innate immunity^17^. Respiratory syncytial virus (RSV) is a member of the *Paramyxoviridae* family, and RSV-expressed nonstructural NS2 protein inhibits IFN transcription induced by binding RIG-I and inhibiting its interaction with the downstream component MAVS^18^. Porcine epidemic diarrhea virus (PEDV) is a member of the *Coronaviridae* family, and PEDV-expressed papain-like protease 2 (PLP2), which has deubiqutinase (DUB) activity, reduces both K48-linked and K63-linked polyubiquitin chains and inhibits RIG-I-activated IFN expression^19^. Herpes simplex virus type 1 (HSV-1)-expressed US11 protein antagonizes IFN-β production by binding RIG-I^20^. Kaposi’s sarcoma-associated herpesvirus (KSHV)-expressed ORF64, which is a tegument protein with DUB activity, suppresses type I IFN signaling by blocking the ubiquitination of RIG-I^21^. Cellular ubiquitin-specific proteases (USPs), a subfamily of DUB^22^, regulate ubiquitination of RIG-I^23^. USP21 and USP15 remove K63-linked polyubiquitin chains from RIG-I and block the ability of RIG-I to induce IFN-β^24^,^25^. USP15 also deubiquitinates the K48-linked ubiquitylation of TRIM25 and facilitates the activation of RIG-I^25^. In addition, the cellular E3 ubiquitin ligase RNF125 mediates K48-linked ubiquitination and destabilization of RIG-I^26^. Protein kinase C-α/β (PKC-α/β) phosphorylates RIG-I and blocks RIG-I-mediated induction of type I IFN^27^. The IFN-inducible protein IFI35 suppresses dephosphorylation and activation of RIG-I and mediates degradation of RIG-I via K48-linked ubiquitination, resulting in blockage of type I IFN induction^28^.

Glioma tumor suppressor candidate region gene 2 protein (GLTSCR2) is a nucleolar protein containing multiple nucleolar localization sequences^29^,^30^. GLTSCR2 was shown to directly interact with viral proteins, such as ICP22 and ICP0 of HSV-1 and KS-Bcl-2 of KSHV in infected cells^31^,^32^. However, it was not clear whether GLTSCR2 might be involved in viral replication. In this work, we pursued the role of GLTSCR2 in viral replication. Translocation from nucleus to cytoplasm enabled GLTSCR2 to attenuate the ability of RIG-I to induce IFN-β in cells responding to viral infection. We also investigated mechanisms for GLTSCR2-induced attenuation of RIG-I. Our studies revealed a previously unrecognized role of GLTSCR2 in attenuation of RIG-I and IFN-β and, for the first time, our results provided insights into nucleolar proteins involved in innate immunity response to viral infection.

## Results

### GLTSCR2 for production of viral particles

**Figure 1:**
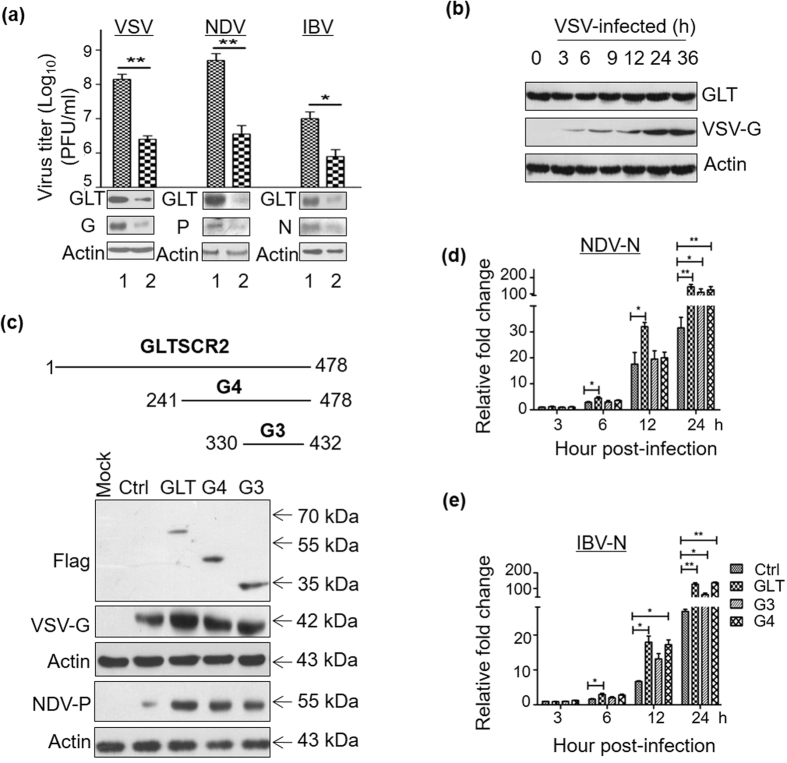
GSTSCR2 in viral replication. (**a**) Cultures of HEp-2 or HEK293T cells in T-25 flask were transfected with control siRNA (200 pmol, lane 1) or GLTSCR2-specific siRNA (200 pmol, lane 2) for 72 h, followed by infection of cells HEK293T with VSV (strain Indiana) at a MOI of 0.1, HEp-2 with NDV (strain F48E9) or IBV (strain M41) at 10 PFU per cell for 48 h. Titers of viruses were measured, as log_10_ PFU/ml. Values represent means of triplicates with standard deviations (SD). Bottom, cell lysates were prepared, GSTSCR2 (GLT) and viral proteins (VSV-G, NDV-N, and IBV-N) were measured by immunoblotting with specific antibodies, with actin as a control. (**b**) HEK293T cells in T-25 flask were infected with VSV at a MOI of 1 for 0, 3, 6, 9, 12, 24, and 36 h. Cell lysates were prepared, GLTSCR2 and VSV-G were measured by immunoblotting with specific antibodies, with actin as a control. (**c**) HEp-2 cells in T-25 flask were transfected with control plasmid (Ctrl) and plasmids to express Flag-tagged wt GLTSCR2 or truncated GLTSCR2 variants G3 and G4 (5 μg each) for 36 h, followed by infection with VSV or NDV at a MOI of 0.1 for 48 h. Cell lysates were analyzed by immunoblotting with specific antibodies to detect Flag, VSV-G, NDV-P, with actin as a control. (**d,e**) HEp-2 cells in T-25 flask were transfected with plasmid vector as control (Ctrl) and plasmids to express Flag-tagged wt GLTSCR2 or truncated G3 and G4 (5 μg each), followed by infection with NDV or IBV at 10 PFU per cell. Total RNAs were isolated; gene expression levels of NDV-N (**d**) and IBV-N (**e**) were quantified by qRT-PCR and normalized with glyceraldehyde-3- phosphate dehydrogenase (GAPDH), the level set in control cells as 1. Values represent means of triplicates with SD. *p < 0.05, **p < 0.01.

To investigate whether GLTSCR2 might play any role in viral replication, we used small interfering RNA (siRNA) to knock down GLTSCR2 in cells, followed by infection with *Rhabdoviridae* vesicular stomatitis virus (VSV), *Paramyxoviridae* Newcastle disease virus (NDV), and *Coronaviridae* infectious bronchitis virus (IBV). As shown in Fig. 1a, decreases of GLTSCR2 resulted in reduced production of viral particles of VSV, NDV, and IBV by ~60, ~120, and ~20 folds, respectively. GLTSCR2 reduction also resulted in decreased levels of viral proteins in cells, indicating an important role of GLTSCR2 for production of various viruses. To investigate if viral infection might modulate GLTSCR2 level in cells, we studied the time-course of GLTSCR2 during VSV infection, and we detected that GLTSCR2 level was not significantly affected by viral infection (Fig. 1b), indicating the endogenous level of GLTSCR2 was sufficient to support viral replication.

Studies indicated that the C-terminal region ranging from residue 347 to 478 contains key nucleolar localization sequences^29^,^30^. The C-terminal region of residues 330 to 478 plays a role in GLTSCR2 interaction with other cellular proteins, such as PTEN^33^,^34^, p53^35^,^36^, and merlin^37^. The region of residues 342 to 386 is also responsible for homo-dimerization or even homo-oligomerization^38^. To understand if these putative domains might be important in GLTSCR2-involved viral replication, we constructed plasmid expression vectors to carry gene fragments encoding G3 (residues 330–432) and G4 (residues 241–478). We transfected HEp-2 cells with these expression vectors individually and infected cells with VSV. We detected that ectopic expression of the full length, wild-type (wt) GLTSCR2 enhanced viral replication, indexed by viral proteins VSV-G and NDV-P (Fig. 1c). Expression of G3 or G4 appeared to enhance viral replication to a comparable level as enhanced by wt GLTSCR2 (Fig. 1c). These preliminary results indicated that the integrity of C-terminal region G3 (residues 330–432) was important for GLTSCR2 in support of viral replication.

To further verify the function of these regions in viral replication, we ectopically expressed wt, G3, and G4 of GLTSCR2 and infected cells with NDV or IBV. As shown in Fig. 1d,e, ectopic expression of wt and variants of GLTSCR2 was able to increase gene expression of viral NDV-N (Fig. 1d) and IBV-N (Fig. 1e), especially in 24 hours after viral infection. Meanwhile, expression of wt GLTSCR2, but not G3 or G4, consistently resulted in upregulation of these viral genes in 6 hours after viral infection and supported viral replication to higher levels than G3 and G4 did throughout the entire infection course. The results lead us to suggest that GLTSCR2 played a supportive role in viral replication.

### Translocation of GLTSCR2 to cytoplasm for VSV replication

**Figure 2:**
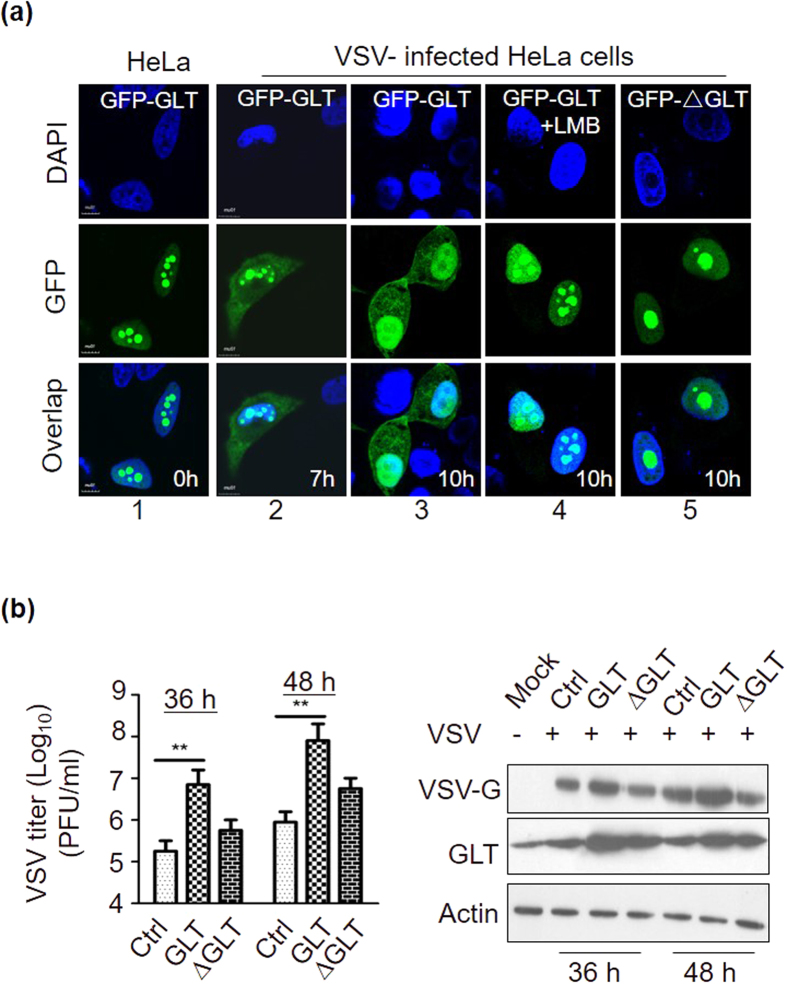
Translocation of GLTSCR2 to cytoplasm for VSV replication. (**a**) HeLa cells grown in 24-well plate were transfected with plasmids to express GFP-tagged GLTSCR2 (lanes 1–4) or NES deletion mutant (ΔGLT, lane 5) (500 ng each) for 24 h, then mock-infected (lane 1) or infected with VSV at a MOI of 5 for 7 h (lane 2). The infected cells were treated with DMSO (lanes 3, 5) or 10 μM of LMB (lane 4) for another 3 h. Then the cells were fixed with 4% paraformaldehyde. Nuclei were stained with DAPI, and GLTSCR2 was visualized from the fused GFP fluorescence. (**b**) HEK293T cells in T-25 flask were transfected with control plasmid, plasmids to express wt GLTSCR2 or ΔGLT (5 μg each), then infected with VSV at a MOI of 0.1 for 36 or 48 h, titers of viruses were measured, as log_10_ PFU/ml. Values represent means of triplicates with SD. Under the same experimental condition, cell lysates were analyzed by immunoblotting with specific antibodies to detect GLTSCR2 and VSV-G, with actin as a control (right). *p < 0.05, **p < 0.01.

GLTSCR2 is a nuclear protein. It was reported that the translocation of GLTSCR2 from nucleus to cytoplasm was induced by infection with HSV-1^32^, however, it was not clear whether GLTSCR2 cytoplasmic translocation might be associated with viral replication. To verify the cytoplasmic translocation of GLTSCR2 in response to VSV replication, we ectopically expressed GFP-tagged GLTSCR2 in HeLa cells, followed by infection of cells with VSV. As shown in Fig. 2a, GFP-tagged GLTSCR2 was detectable in the nucleus (lane 1), and then was detected in the cytoplasm by 7 (lane 2) and 10 (lane 3) hours after viral infection. Cytoplasmic translocation of GFP-tagged GLTSCR2 was blocked by leptomycin B (LMB) (lane 4), which is able to inhibit broad range of nuclear protein export pathways mediated by CRM1/exportin 1^39^, indicating that the export of GLTSCR2 to cytoplasm was in a CRM1-dependent manner. In addition, GLTSCR2-∆GLT variant, in which the nuclear export sequence (NES, residues 358 to 370) was deleted, failed to translocate into the cytoplasm after VSV infection (lane 5). Furthermore, the levels of viral production (Fig. 2b, left) and viral proteins (Fig. 2b, right) were greatly increased by ectopic expression of wt GLTSCR2. The ectopic expression of GLTSCR2-∆GLT, however, resulted in slightly increased production of viral particles (Fig. 2b). The results indicated that cytoplasmic translocation of GLTSCR2 played an important role in support of VSV replication.

### GLTSCR2 attenuated IFN-β

**Figure 3:**
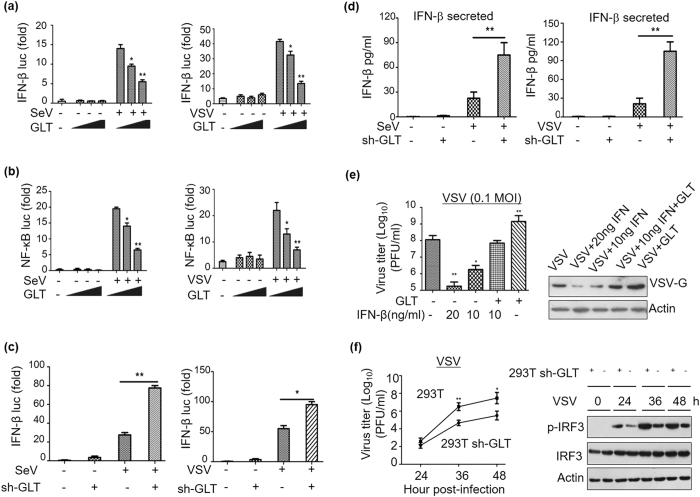
GLTSCR2 attenuates IFN-β. (**a,b**) HEK293T cells in 24-well plate were co-transfected with control plasmid (100 ng) or increasing doses of plasmids to express GLTSCR2 (50, 100, 200 ng), along with 2 ng pRL-TK and 200 ng IFN-β-Luc (**a**) or NF-κB-Luc (**b**) for 48 h. The cells were infected with SeV at 50 HA U/ml (left) or VSV at a MOI of 0.1 (right) for 12 h, and used for dual-luciferase reporter assays. Values of IFN-β reporter activity presented in all the promoter reporter assays are expressed as relative fold changes over the mock-infected control cells. Values represent means of triplicates with SD. (**c**) HEK293T cells in 24-well plate were transfected with pGMLV-SC1 vector encoding control shRNA (−) or GLTSCR-specific shRNA (+) for 72 h, then assessed as described in (**a**). (**d**) HEK293T cells in 24-well plate were transfected with pGMLV-SC1 vector encoding control shRNA (−) or shRNA targeting GLTSCR2 (+) for 72 h, mock-infected or infected with SeV at 50 HA U/ml (left) or VSV at a MOI of 0.1 (right) for 12 h, the supernatants from cells were harvested and analyzed for IFN-β production by ELISA. Values represent means of triplicates with SD. (**e**) HEK293T cells in T-25 flask transfected with control plasmid or Flag-tagged GLTSCR2 (5 μg each) were treated with commercially recombinant IFN-β at concentrations of 10 or 20 ng/ml, followed by infection of cells with VSV at a MOI of 0.1 for 48 h, the virus yields were determined (left). Values represent means of triplicates with SD. Under the same experimental condition, electrophoretically separated proteins were analyzed by immunoblotting with specific antibody to detect VSV-G, with actin as a control (right). (**f**) HEK293T or GLTSCR2 knockdown cells (293T sh-GLT) cells in T-25 flask were infected with VSV at a MOI of 0.1. Titers of viruses were measured, as log_10_ PFU/ml. Values represent means of triplicates with SD. Under the same experimental condition, electrophoretically separated proteins from HEK293T (−) or 293T sh-GLT (+) cells infected with VSV were analyzed by immunoblotting with specific antibodies to detect p-IRF3, IRF3, with actin as a control (right). *p < 0.05; **p < 0.01.

To reveal the mechanism for GLTSCR2 in supporting viral replication, we investigated any effects of ectopically expressed GLTSCR2 on IFN-β. After viral infection, increases of ectopically expressed GLTSCR2 resulted in decreases of IFN-β promoter activity in cells infected with either Sendai virus (SeV) or VSV (Fig. 3a). We also detected that increases of ectopically expressed GLTSCR2 resulted in decreasing NF-κB-dependent promoter activity in cells infected with either SeV or VSV (Fig. 3b). These results indicated that GLTSCR2 might be involved in downregulation of IFN-β and NF-κB during viral infection of cells. In contrast, knockdown of endogenous GLTSCR2 resulted in significant increases of IFN-β promoter activity (Fig. 3c) and IFN-β secretion (Fig. 3d) in cells after infection with SeV or VSV. These results verified the ability of GLTSCR2 to mediate downregulation of IFN-β during viral infection.

In addition, the ability of recombinant IFN-β to suppress VSV replication, indexed by viral particle production (Fig. 3e, left) and the viral protein VSV-G (Fig. 3e, right), was significantly reversed by ectopically expressed wt GLTSCR2. The results further indicated that GLTSCR2 supported viral replications by not only downregulating IFN-β expression but also suppressing IFN-β activity. Phosphorylation of type I IFN regulatory factor IRF3 on serine 396 has been shown to play a pivotal role in IFN-β induction^10^,^11^. While investigating the involvement of IRF3 activation in GLTSCR2 suppression of type I IFN, we generated HEK293T cell line stably expressing specific short hairpin RNA (shRNA) targeting GLTSCR2 using a shRNA-based lentivector (GLTSCR2 knockdown cells). Our results showed that VSV replication was decreased in GLTSCR2 knockdown cells versus viral replication in HEK293T cells (Fig. 3f, left). We also detected that a higher level of phosphorylated IRF3 (p-IRF3) in GLTSCR2 knockdown cells than it in HEK293T cells infected with VSV (Fig. 3f, right) for 24, 36, and 48 hours. These results indicated that p-IRF3 reduction was involved in GLTSCR2 mediated suppression of IFN-β induction in the support of viral replication.

### GLTSCR2 targeted and blocked RIG-I activity

**Figure 4:**
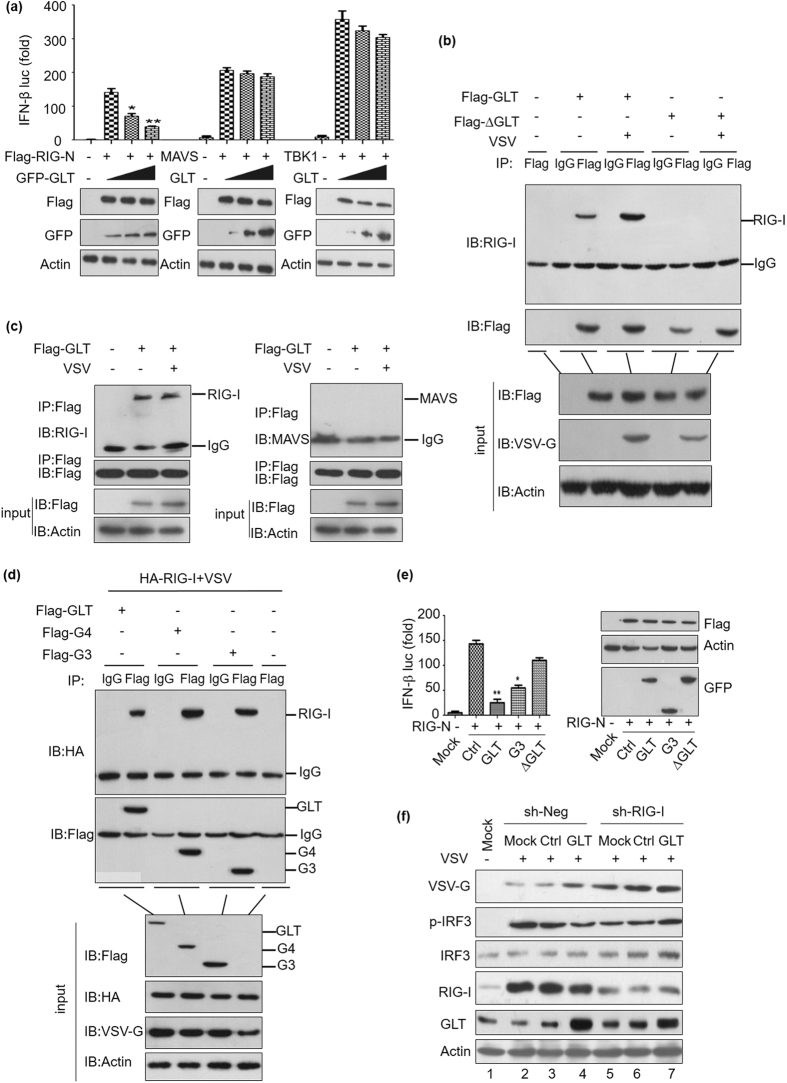
GLTSCR2 downregulates RIG-I. (**a**) HEK293T cells were co-transfected with increasing doses of plasmids to express GFP-tagged GLTSCR2 (50, 100, 200 ng) and Flag-tagged RIG-I-N, MAVS, or TBK1 (200 ng each), along with IFN-β-Luc and pRL-TK for 48 h. Dual-luciferase reporter assays were performed, and assessed as described in Fig. 3(a). Bottom, electrophoretically separated proteins were analyzed by immunoblotting with the indicated antibodies, with actin as a control. (**b**) HEK293T cells in T-25 flask were transfected with plasmids to express Flag-tagged GLTSCR2 or Flag-tagged ΔGLT (5 μg each) for 36 h, then mock- infected or infected with VSV at a MOI of 0.1 for 12 h. Cell lysates were immunoprecipitated with antibody to Flag or IgG (2–3 μg). The immuno- precipitates were analyzed by immunoblotting with the indicated antibodies. Expression of proteins from the transfected plasmids was analyzed by immunoblotting with the indicated antibodies, with actin as a control (input). (**c**) Under the experimental condition same with (**b**), cell lysates were immunoprecipitated with antibody to Flag. The immuno- precipitates were analyzed by immunoblotting with specific antibodies to detect RIG-I (left) and MAVS (right). (**d**) HEK293T cells in T-25 flask were co-transfected with plasmids to express HA-tagged RIG-I and Flag-tagged GLTSCR2, or truncated G3 and G4 (5 μg each) for 36 h, then infected with VSV for 12 h. Cell lysates were immunoprecipitated with antibody to Flag or IgG, and the immuno- precipitates were analyzed by immunoblotting with the indicated antibodies. (**e**) HEK293T cells were co-transfected with plasmids to express Flag-tagged RIG-I-N and GFP-tagged GLTSCR2, G3, ΔGLT, or control, along with IFN-β-Luc and pRL-TK for 48 h. The cells were used for dual-luciferase reporter assays, and assessed as described in Fig. 3(a). Right, electrophoretically separated proteins were analyzed by immunoblotting with the indicated antibodies. (**f**) HEK293T cells in T-25 flask were transfected with psiRNA vector encoding control shRNA or RIG-I-specific shRNA for 72 h, followed by transfection of control plasmid or Flag-tagged GLTSCR2 for 24 h, then mock-infected or infected with VSV for 24 h. Electrophoretically separated proteins were analyzed by immunoblotting with the indicated antibodies.

To reveal the mechanism for GLTSCR2 in downregulation of the type I IFN, we investigated modulators involved in the regulation of IFN-β. It has been indicated that RIG-I, MAVS, and TBK1 are activated in cells responding to VSV infection^40^. We found that ectopic expression of RIG-I-N (constitutively-active RIG-I variant consisting of residues 1–300), MAVS, or TBK1 was able to induce IFN-β promoter activity, and co-expression of GLTSCR2 blocked IFN-β promoter activation by RIG-I-N but not MAVS or TBK1 (Fig. 4a), indicating the ability of GLTSCR2 to block RIG-I.

Then, we investigated whether GLTSCR2 might interact with RIG-I in downregulation of IFN-β. We used Flag-specific antibodies to immune-precipitate ectopically-expressed Flag-tagged wt GLTSCR2 or GLTSCR2-∆GLT variant and used specific antibodies in immunoblotting to detect if endogenous RIG-I was in the immune complexed in cells with and without being infected with VSV. The result revealed that GLTSCR2 interacted with RIG-I. The NES-deletion mutant ΔGLT of GLTSCR2, however, failed to interact with RIG-I, suggesting that the deletion of the sequences 358–370 disrupted their interaction (Fig. 4b). Under the same experimental condition, we found that RIG-I (Fig. 4c, left), but not MAVS (Fig. 4c, right), was in the immune complexes with GLTSCR2 regardless of viral infection. In addition, HA-tagged RIG-I was detected in the immune-complexes with G3 and G4 variants versus wt GLTSCR2 (Fig. 4d). Apparently, the C-terminal region of GLTSCR2 was able to interact with RIG-I. However, whether the N-terminal region of GLTSCR2 was involved in interaction with RIG-I remains to be clarified.

Furthermore, co-expression of wt GLTSCR2 and G3, but not the NES-deletion mutant ΔGLT, blocked RIG-I-N-induced IFN-β promoter activation; and G3 was less effective than wt GLTSCR2 in the blockage of RIG-I-N-induced IFN-β promoter activation (Fig. 4e). These results taken together indicated that C-terminal region G3 (residues 330–432) was able to interact with RIG-I for blocking the ability of RIG-I to induce IFN-β, and the cytoplasmic translocation of GLTSCR2 were important in downregulation of IFN-β expression.

To further explore whether GLTSCR2 blocked the ability of RIG-I by directly targeting RIG-I, HEK293T or RIG-I knockdown cells were transfected with Flag-tagged GLTSCR2. Our results revealed that the viral protein level increased, while p-IRF3 level decreased in RIG-I knockdown cells (Fig. 4f, lanes 5–7) as compared with that in HEK293T cells (lanes 2–4). Ectopically expression of GLTSCR2, however, failed to support viral replication (lanes 5–7). This result indicated that GLTSCR2 blocked the ability of RIG-I to induce IFN-β that supports viral replication.

### GLTSCR2 negatively regulated RIG-I via K63-linked ubiquitination

**Figure 5:**
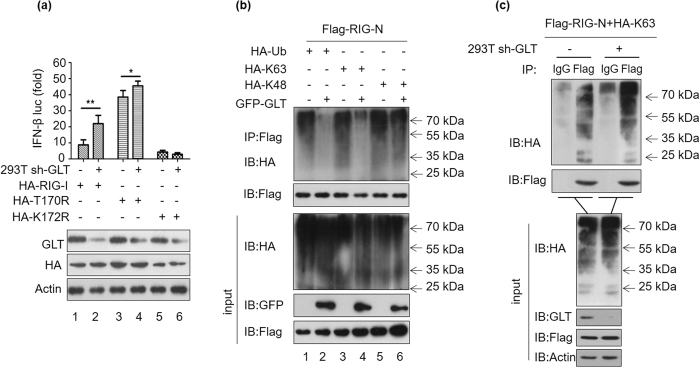
GLTSCR2 de-conjugates K63-linked polyubiquitin chains from RIG-I. (**a**) HEK293T cells (lanes 1, 3, 5) or 293T sh-GLT cells (lanes 2, 4, 6) in 24-well plate were transfected with plasmids to express 200 ng IFN-β-Luc, 2 ng pRL-TK and HA-tagged wt RIG-I (lanes 1, 2), or variants T170R (lanes 3, 4) and K172R (lanes 5, 6) (200 ng each), followed by infection of cells with VSV at a MOI of 0.01 for 12 h. Reporter assays were performed at 24 h after transfection. Values represent means of triplicates with SD. Bottom, electrophoretically separated proteins were analyzed by immunoblotting with specific antibodies to detect GLTSCR2, or HA-tagged RIG-I, T170R and K172R, with actin as a control. (**b**) HEK293T cells in T-25 flask were co-transfected with plasmids to express Flag-tagged RIG-I-N and control (lanes 1, 3, 5) or GFP-tagged GLTSCR2 (lanes 2, 4, 6) (5 μg each), along with HA-tagged ubiquitin (lanes 1, 2), K63-linked ubiquitin (lanes 3, 4), or K48-linked ubiquitin (lanes 5, 6) (5 μg each). Cell lysates were immunoprecipitated with antibody to Flag. The immuno- precipitates were analyzed by immunoblotting with the indicated antibodies. (**c**) HEK293T cells (−) or 293T sh-GLT cells (+) in T-25 flask were co-transfected with plasmids to express Flag-tagged RIG-I-N and HA-tagged K63-linked ubiquitin (5 μg each) for 36 h. Cell lysates were immunoprecipitated with antibody to Flag or IgG (2–3 μg). The immuno- precipitates were analyzed by immunoblotting with the indicated antibodies. *p < 0.05; **p < 0.01.

It has been shown that the dephosphorylation (threonine T170) and K63-linked ubiquitination (lysine K172) lead to RIG-I activation and IFN-β production^2^,^13^,^26^. To address whether these sites in RIG-I were also critical for interaction with GLTSCR2, we substituted arginine (R) for T170 and K172 to arginine (R) to result in RIG-I-T170R and RIG-I-K172R variants, respectively, afterthen ectopically expressed these RIG-I variants in HEK293T or GLTSCR2 knockdown cells. We detected that ectopic expression of wt RIG-I induced IFN-β promoter activity (Fig. 5a, lane 1), and knockdown of GLTSCR2 resulted in increasing wt RIG-I induced IFN-β promoter activity (lane 2). Expression of RIG-I-T170R significantly increased IFN-β promoter activity to a higher level than wt RIG-I induced (lane 3), and knockdown of GLTSCR2 also increased RIG-I-T170R-induced IFN-β promoter activity (lane 4). Expression of RIG-I-K172R induced a lower level of IFN-β promoter activity than wt induced (lane 5), and knockdown of GLTSCR2 failed to affect RIG-I-K172R-induced IFN-β promoter activity (lane 6). The results showed that the two sites of T170 and K172 of RIG-I were critically involved in induction of IFN-β promoter activity, in line with the results reported by Wiels and Gack (lanes 1, 3, 5). Furthermore, knockdown of GLTSCR2 potentiated wt RIG-I or mutant T170R induced activation of the IFN-β promoter, as shown in Fig. 5a, lanes 2, 4. It can be excluded that GLTSCR2 targeted RIG-I via dephosphorylation of RIG-I.

To further study whether GLTSCR2 modulated ubiquitination of RIG-I, HEK293T cells were co-transfected with plasmids to express Flag-tagged RIG-I-N and GFP-tagged GLTSCR2, along with HA-tagged ubiquitin, K63-linked ubiquitin, or K48-linked ubiquitin. We detected that ectopic expression of GLTSCR2 was able to induce de-conjugation of K63-linked (Fig. 5b, lane 4), but not K48-linked (lane 6) polyubiquitin chains from the constitutively-active variant RIG-I-N. Knockdown of endogenous GLTSCR2 resulted in increased K63-linked ubiquitination of RIG-I-N (Fig. 5c). These results taken together indicated that GLTSCR2 was able to downregulate RIG-I, via K63-linked ubiquitination.

### USP15 mediated GLTSCR2 removal of RIG-I ubiquitination

**Figure 6:**
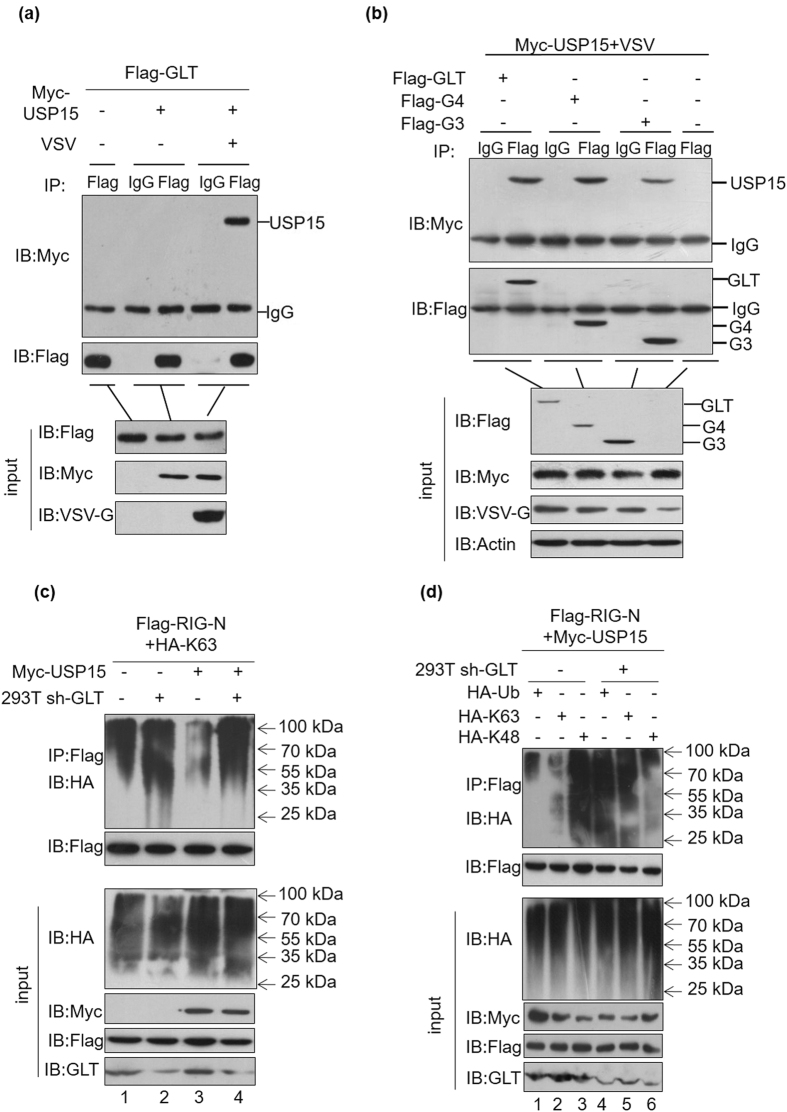
USP15 mediates GLTSCR2 removal of RIG-I ubiquitination. (**a**) HEK293T cells in T-25 flask were co-transfected with plasmids to express Flag-tagged GLTSCR2 (5 μg) and Myc-tagged USP15 (5 μg) for 36 h, then mock- infected or infected with VSV at a MOI of 0.1 for 12 h. Cell lysates were immunoprecipitated with antibody to Flag or IgG (2–3 μg). The immuno- precipitates were analyzed by immunoblotting with the indicated antibodies. (**b**) HEK293T cells in T-25 flask were co-transfected with plasmids to express Myc-tagged USP15 (5 μg) and Flag-tagged GLTSCR2, G3, or G4 (5 μg each) for 36 h, then infected with VSV at a MOI of 0.1 for 12 h. Cell lysates were immunoprecipitated with antibody to Flag or IgG (2–3 μg). The immuno- precipitates were analyzed by immunoblotting with the indicated antibodies. (**c**) HEK293T cells (lanes 1, 3) or 293T sh-GLT cells (lanes 2, 4) in T-25 flask were co-transfected with plasmids to express Flag-tagged RIG-I-N (5 μg) and HA-tagged K63-linked ubiquitin (5 μg), along with control (lanes 1, 2) or Myc-tagged USP15 (lanes 3, 4) (5 μg each). Cell lysates were immunoprecipitated with antibody to Flag (2–3 μg). The immuno- precipitates were analyzed by immunoblotting with the indicated antibodies. (**d**) HEK293T cells (lanes 1, 2, 3) or 293T sh-GLT cells (lanes 4, 5, 6) were co-transfected with Flag-tagged RIG-I-N and Myc-tagged USP15, along with HA-tagged ubiquitin (lanes 1, 4), K63-linked ubiquitin (lanes 2, 5), or K48-linked ubiquitin (lanes 3, 6). Cell lysates were immunoprecipitated with antibody to Flag. The immuno-precipitates were analyzed by immunoblotting with the indicated antibodies.

USP15 has been shown to remove K63-linked, but not K48-linked, polyubiquitin chains from RIG-I, resulting in deactivation of RIG-I and blockage of IFN-β induction^24^. Addressing whether USP15 was responsible for GLTSCR2-induced K63-linked ubiquitination of RIG-I, we ectopically co-expressed Myc-tagged USP15 and Flag-tagged GLTSCR2 in cells with and without being infected with VSV. The result showed that USP15 can interact with GLTSCR2 upon viral infection (Fig. 6a). Furthermore, we detected that USP15 was in the immune complexes with wt, G3, and G4 variants of GLTSCR2, at the moment of VSV infection (Fig. 6b). These results indicated that USP15 interacted with GLTSCR2, via binding to the range of residues 330–432 (G3).

To address whether USP15 was responsible for GLTSCR2 regulation of K63-linked ubiquitination of RIG-I, we co-expressed Flag-tagged RIG-I-N and HA-tagged K63 ubiquitin in the presence or absence of Myc-tagged USP15 in HEK293T or GLTSCR2 knockdown cells. We detected that K63-linked ubiquitination of RIG-I-N was increased in GLTSCR2 knockdown cells (Fig. 6c, lane 2) versus in HEK293T cells (lane 1) in the absence of USP15. Presence of USP15 reduced K63-linked ubiquitination of RIG-I-N in HEK293T cells (lane 3); in contrast, USP15 failed to reduce K63-linked ubiquitination of RIG-I-N in GLTSCR2 knockdown cells (lane 4). The results indicated that presence of GLTSCR2 was required for USP15 to effectively remove K63-linked polyubiquitin chains from RIG-I. Interestingly, we detected that reduction of endogenous GLTSCR2 resulted in increased USP15 activity in removing K48-linked ubiquitination of RIG-I-N in GLTSCR2 knockdown cells (Fig. 6d, lane 6), whereas GLTSCR2 presence reduced the activity of USP15 in removing K48-linked ubiquitination of RIG-I-N in HEK293T cells (lane 3). It appeared that GLTSCR2 supported and inhibited the ability of USP15, respectively, to remove K63-linked and K48-linked ubiquitination of RIG-I. These results taken together indicated that GLTSCR2 interacted with RIG-I and USP15 in a complex to support the activity of USP15 to remove K63-linked polyubiquitin chains from RIG-I, leading to inactivation of RIG-I and blockage of IFN-β induction.

**Figure 7:**
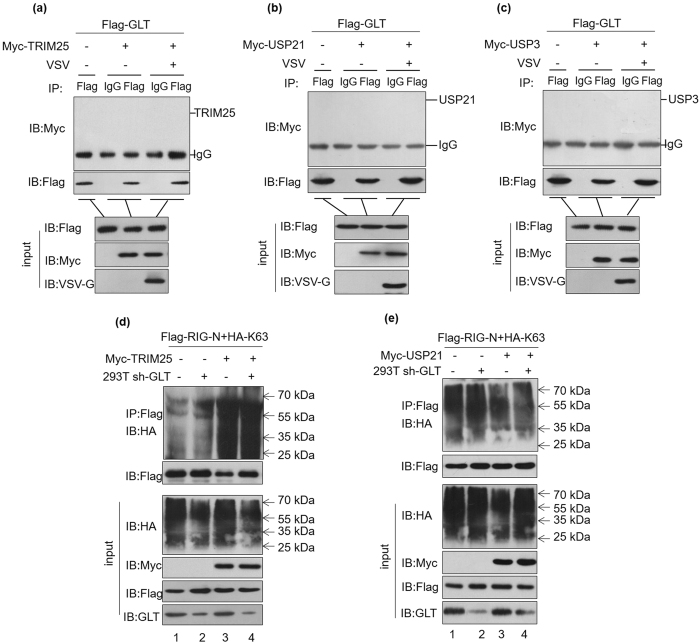
TRIM25, USP21, USP3 are not involved in GLTSCR2 regulating RIG-I ubiquitination. (**a–c**) HEK293T cells in T-25 flask were co-transfected with plasmids to express Flag-tagged GLTSCR2 (5 μg) and Myc-tagged TRIM25 (**a**), USP21 (**b**), or USP3 (**c**) (5 μg each) for 36 h, then mock-infected or infected with VSV at a MOI of 0.1 for 12 h. Cell lysates were immunoprecipitated with antibody to Flag or IgG (2–3 μg). The immuno- precipitates were analyzed by immunoblotting with the indicated antibodies. (**d,e**) HEK293T cells (lanes 1, 3) or 293T sh-GLT cells (lanes 2, 4) in T-25 flask were co-transfected with plasmids to express Flag-tagged RIG-I-N (5 μg) and HA-tagged K63-linked ubiquitin (5 μg), along with Myc-tagged TRIM25 (**d**) or Myc-tagged USP21 (**e**) (5 μg each). Cell lysates were immunoprecipitated with antibody to Flag (2–3 μg). The immuno- precipitates were analyzed by immunoblotting with the indicated antibodies.

Attachment of K63-linked polyubiquitin chains to RIG-I by the ubiquitin E3 ligase TRIM25 leads to the activation of RIG-I^26^. USP21 and USP3 have also been shown to remove K63-linked polyubiquitin chains from RIG-I, resulting in deactivation of RIG-I^24^,^41^,^42^. Here, we ectopically co-expressed Flag-tagged GLTSCR2 and Myc-tagged TRIM25 (Fig. 7a), USP21 (Fig. 7b), or USP3 (Fig. 7c) in cells with and without being infected with VSV to detect if they were in the immune complexed with GLTSCR2, under the experimental condition similar to Fig. 6a. It was showed no evidence of the interaction regardless of viral infection. To further address whether TRIM25 or USP21 were responsible for GLTSCR2 regulation of K63-linked ubiquitination of RIG-I, we co-expressed Flag-tagged RIG-I-N and HA-tagged K63 ubiquitin in the presence or absence of TRIM25 (Fig. 7d) or USP21 (Fig. 7e) in HEK293T or GLTSCR2 knockdown cells. No evidence was presented to support TRIM25 or USP21 mediated GLTSCR2 removal of K63-linked polyubiquitin chains from RIG-I.

## Discussion

In this work, we presented evidence that viral infection induced translocation of GLTSCR2 from nucleus to cytoplasm, and cytoplasmic translocation enabled GLTSCR2 to effectively attenuate IFN-β and support viral replication; however, viral infection did not result in elevating GLTSCR2 in cells. It was reported that viruses are able to utilize host cell’s ubiquitin ligase and deubiqutinases to antagonize RIG-I-, MAVS-, TBK1-, and IRF3-mediated innate immune response in order to succeed viral replications^43^,^44^,^45^. Cellular proteins, such as PCBP2, Ndfip1, SOCS3, Siglec1, and FoxO1, are involved in recruiting ubiquitin ligases to down-regulate MAVS, TBK1, and IRF3 by K48-linked ubiquitination and degradation^46^,^47^,^48^,^49^,^50^. On the other hand, studies showed that influenza A virus infection induces nuclear accumulation of β-catenin to suppress IFN-β induction^51^, and nuclear translocation of TRIM26 promotes K48-linked ubiquitination and degradation of IRF3, thereby blocking IFN-β induction^43^. In contrast, our studies showed that cytoplasmic translocation of nucleolar GLTSCR2 contributed to attenuation of IFN-β induction and replication of viral particles. Abrogating the cytoplasm-translocating ability of GLTSCR2 diminished its ability to attenuate IFN-β induction and support viral replication. Accordingly, targeting cytoplasmic translocation of GLTSCR2 should be seriously considered in the development of antiviral agents.

RIG-I, which acts as a sensor to recognize viral infection and intracellular infection of bacteria, plays an important role in the induction of type I IFN for cellular defense^5^,^52^,^53^. The amino-terminal caspase recruitment domains (CARDs) of RIG-I (RIG-N) undergo robust ubiquitination induced by TRIM25^14^ and TRIM4^15^, which effectively deliver the Lys 63-linked polyubiquitin chains to the RIG-N, to increase the activity of RIG-I. However, an ubiquitin-specific proteases USP15^24^, USP21^41^, or USP3^42^, removes the 63-linked polyubiquitin chains from the RIG-N to attenuate the activity of RIG-I. K63-linked ubiquitination of RIG-I results in activation of RIG-I, leading to induction of type I IFN^14^, whereas K48-linked ubiquitination results in inactivation of RIG-I^26^. GLTSCR2 was able to interact with RIG-I and abolish the ability of RIG-I to induce IFN-β, via de-conjugation of K63-linked, but not K48-linked, polyubiquitin chains from RIG-I. Thus, RIG-I played an important role as an immediate downstream target of GLTSCR2 for inactivation in attenuation of type I IFN and facilitation of viral replication.

USP15 has been shown to remove K63-linked polyubiquitin chains from RIG-I, resulting in deactivation of RIG-I^24^. We detected that USP15, but not USP3 or USP21, involved in GLTSCR2-induced removal of K63-linked polyubiquitin chains from RIG-I, resulting in deactivation of RIG-I and blockage of IFN-β induction. Interestingly, decreases of endogenous GLTSCR2 resulted in increasing the ability of USP15 to remove K48-linked ubiquitination of RIG-I. Possibly, K63-linked and K48-linked ubiquitination competed for the same residue K172 in RIG-I, and a threshold of GLTSCR2 level played the role of modulating USP15 activity to de-conjugate K63-linked or K48-linked ubiquitination. It is also possible that cytoplasmic translocation increased GLTSCR2 level to induce the ability of USP15 to de-conjugate K63-linked ubiquitination of RIG-I; in contrast, lack of cytoplasmic GLTSCR2 resulted in decreasing the ability of USP15 to de-conjugate K63-linked ubiquitination of RIG-I but increasing the USP15 ability to de-conjugate K48-linked ubiquitination of RIG-I. Decreases of K63-linked ubiquitination or increases of K48-linked ubiquitination of RIG-I resulted in attenuation of RIG-I, thereby blocking the RIG-I to IFN-β pathway and supporting viral replication.

Type I IFN plays an important role in host cell defense against viral infection. Our studies revealed a novel role the nucleolar protein GLTSCR2 played in attenuation of IFN-β, via cytoplasmic translocation to induce the ability of USP15 to deactivate RIG-I. Our findings advanced current knowledge of nucleolar modulators, translocated into the cytoplasm, for attenuation of type I IFN and support viral replication during viral infection. However, whether cytoplasmic translocation of GLTSCR2 and abrogation of RIG-I activity might induce additional pathways, bypassing attenuation of IFN-β, to support viral replication remains to be clarified. What viral components or cellular modulators, in response to viral infection, induced GLTSCR2 translocation to the cytoplasm also needs to be studied.

### Materials and Experimental procedures

#### Cells

HEp-2, HeLa, and HEK293T cells were cultured in Dulbecco’s modified Eagle’s medium (DMEM) with high glucose that was supplemented with 2 mM L-glutamine, nonessential amino acids, sodium pyruvate, and 10% heat-inactivated fetal bovine serum (FBS), 100 U/ml penicillin and 100 μg/ml streptomycin (all reagents purchased from Gibco Invitrogen). All cells were cultured at 37 °C in a humidified incubator with 5% CO_2_.

#### Antibodies

Rabbit polyclonal antibody to GLTSCR2 (used at 1:1000), mouse monoclonal antibody RIG-I and MAVS (used at 1:800) were acquired from abcam. The anti VSV-G (used at 1:200) mouse monoclonal antibody and the rabbit antibody to the nuclear protein histone H3 (used at 1:100) were obtained from Santa Cruze. The antibody to HA (used at 1:1000), Flag (used at 1:800), Myc (used at 1:800), actin (used at 1:1000), and GFP (used at 1:800) were obtained from Beyotime Biotechnology. The rabbit polyclonal antibodies to IRF3 (used at 1:600), p-IRF3 (S396, used at 1:600) were purchased from Cell Signaling Technology. The antibodies to NDV-P (used at 1:400) and IBV-N (used at 1:400) were preserved in our laboratory.

#### Knockdown of GLTSCR2 with siRNA and shRNA

Cells were transfected with siRNA targeting GLTSCR2 (Santa), or with pGMLV-SC1 vector encoding GLTSCR-specific shRNA, by means of Lipofectamine^TM^ 2000 according to manufacturer’s protocols. The shRNA target sequences used in this paper was 5′-GCT GAC AAA GAA GAG AAC CAA TTC AAG AGA TTG GTT CTC TTC TTT GTC AGC TTT TTC CAT GG-3′.

#### Generation of knockdown cell line

The primers were designed based on GLTSCR2 coding sequence described in the NCBI database (http://www.ncbi.nlm.nih.gov/protein/AIC56320.1). The shRNA target sequence was inserted at *Xho* I-*EcoR* I sites in the vector. The stable knockdown cells of GLTSCR2 were generated using a shRNA-based lentivector system pSIH-H1 (System Biosciences) as suggested in the instruction manual. The efficiency of knockdown was confirmed by standard qRT-PCR.

#### Plasmids

The vectors pcDNA3.1, pFLAG -cmv3, and pEGFP-N1 were purchased from Clontech. GLTSCR2 and its NES mutant (ΔGLT), and truncated variants including G3 and G4, were cloned in vector using specific primers. Plasmids pcDNA3.1- HA-tagged Full-length Ub, HA-tagged Ub mutants, in which all but one Lys residue (HA-K48 or HA-K63) was replaced with Arg, pcDNA3.1- Myc-tagged USP15, TRIM25, USP21, USP3 were obtained from Shanghai Generay Biotech. The plasmids for Flag-tagged RIG-I and HA-tagged RIG-I were kindly provided by Dan-Ying Chen at Peking University, its constitutively active mutant (Flag-RIG-I-N) was cloned in pFLAG-cmv3 using specific primers. The pIFN-β-Luc reporter for IFN-β, the pNF-κB-Luc reporter for NF-κB, and the internal control plasmid pRL-TK were purchased from Promega. Eukaryotic expression plasmids containing the genes were transfected into cells with the aid of Lipofectamine^TM^ 2000.

#### Plaque formation assay

Cells were mock-infected or exposed to viruses in mixture supplemented with DMEM. After 90 min the inoculum was replaced with 2% FBS of DMEM. Yields of infectious virus were determined by plaque assay for 48 h. Following incubation, plaque numbers were counted. Data represent the mean of three independent experiments.

#### Preparation of cell lysates and immunoblots

The cells were collected by centrifugation, rinsed with phosphate-buffered saline (PBS) containing protease inhibitor cocktail (Roche) and dissolved in 200 μl lysis buffer (pH7.5 20 mM Tris-HCl, 150 mM NaCl, 1% Triton X-100, 1 mM EDTA, 2.5 mM Sodium pyrophosphate, 1 mM β-Glycerrophosphate, 1 mM NaVO4, 1 μg/ml Leupeptin) in the presence of the protease inhibitor cocktail and finally disrupted by sonication. Solubilization of protein was harvested from cells, electrophoresis in denaturing polyacrylamide gels were transferred to polyvinylidene fluoride (PVDF) and reacted with appropriate antibodies. The protein bands were detected with secondary antibodies conjugated to HRP, and actin expression was used as a loading control.

#### Quantitave real-time PCR (qRT-PCR)

Replicate cultures were harvested and total RNA was extracted with Trizol (Invitrogen). A two-step qRT-PCR (SYBR Green I technology, Applied Roche) was performed using SYBR green supermix (Toyobo) according to the manufacturer’s protocol to measure transcription levels for several genes of interest. The primers used were as follow. GAPDH: 5′-CTG GTG ACC CGT GCT GCT T-3′ (forward), 5′-TTG CCG CCT TCT GCC TTA-3′ (reverse). NDV-N: 5′-TGA TGA CCC AGA AGA TAG ATG GAG-3′ (forward), 5′-CTG TGA GTG GGA GCA TAA AAG AGA-3′ (reverse). IBV-N: 5′-GGT TGC TGCT AAG GGT GC-3′ (forward), 5′-GCC TTT GTA ATG CGG GAG-3′ (reverse). Relative fold changes were automatically calculated by the Step One Plus real-time PCR system software (Applied Biosystems), following the ΔΔC_T_ method. GAPDH was also determined and used as internal control.

#### Promoter-driven reporter gene assays

HEK293T cells were transfected with 200 ng of IFN-β-luc, or NF-κB-luc (firefly luciferase) and 2 ng pRL-TK (renilla luciferase plasmid) plasmid for 48 h. Subsequently, cells were infected with SeV or VSV for 12 h before harvesting. Cell lysates were subjected to luciferase assay using a dual-luciferase reporter assay kit (Promega). Luciferase activity was measured using a 20/20^n^ luminometer (Turner Biosystems) and expressed as the relative fold induction (*n*-fold) over the level of activity in the negative control after normalization to the renilla luciferase activity.

#### ELISA for IFN-β

To measure the IFN-β secreted, HEK293T cells were transfected with pGMLV-SC1 vector encoding control shRNA or GLTSCR-specific shRNA for 72 h, then mock-infected or infected with SeV or VSV for 12 h. The supernatants were harvested for ELISA (PBL Biomedical Laboratories) with a commercial sandwich kit (TFB), according to the manufacturer’s instructions.

#### Immunoprecipitation

HEK293T cell cultures were washed 3 times with PBS and resuspended in immunoprecipitation buffer (Applygen Technologies, Inc.) in the presence of a protease inhibitor cocktail, then disrupted by sonication, and clarified by centrifugation at 6,000 for 15 min. The supernatant was then transferred to fresh tubes. A corresponding antibody anti-Flag was added to the cell supernatant and incubated at 4 °C for 12 h. The immune complexes were captured with protein A/G conjugated to beads for 4 h. The beads were rinsed five times with the lysis buffer. The bound proteins were denatured in 2× loading buffer and separated on SDS-PAGE gels, and analyzed by immunoblotting.

## Additional Information

**How to cite this article**: Wang, P. *et al*. The nucleolar protein GLTSCR2 is required for efficient viral replication. *Sci. Rep.* **6**, 36226; doi: 10.1038/srep36226 (2016).

**Publisher’s note:** Springer Nature remains neutral with regard to jurisdictional claims in published maps and institutional affiliations.
